# Two new species of Capitellidae (Annelida, Polychaeta) from the southern coasts of China

**DOI:** 10.3897/zookeys.1292.203560

**Published:** 2026-09-16

**Authors:** Jun-Hui Lin, María E. García-Garza, Shu-Yi Zhang, He-Shan Lin

**Affiliations:** 1 Third Institute of Oceanography, Ministry of Natural Resources, No. 178 Daxue Road, Xiamen 361005, China Third Institute of Oceanography, Ministry of Natural Resources Xiamen China; 2 Universidad Autónoma de Nuevo León, Facultad de Ciencias Biológicas, Laboratorio de Biosistemática, Apartado Postal 5 “F”, San Nicolás de los Garza, Nuevo León, Mexico Universidad Autónoma de Nuevo León, Facultad de Ciencias Biológicas, Laboratorio de Biosistemática San Nicolás de los Garza Mexico

**Keywords:** *
Barantolla
*, East China Sea, molecular sequences, *
Parheteromastus
*, Polychaeta, South China Sea, taxonomy

## Abstract

The annelid family Capitellidae Grube, 1862 remains poorly known in China, with only a few taxa reported to date. Based on specimens collected from coastal zones during recent ecological surveys, we describe two new species belonging to two small genera of Capitellidae. *Barantolla multibranchia***sp. nov**. is superficially similar to *B. sculpta* Southern, 1921 in bearing dorsal branchiae on the posterior abdomen, a complete chaetiger 1, and an areolated epithelium on the anterior thorax, but it differs from the latter in having mixed chaetae in both rami of chaetiger 6 and in lacking cuticular sculpturing. *Parheteromastus simplex***sp. nov**. represents the second species in this small genus. It resembles its congener in lacking branchiae and in having reduced parapodial lobes in the abdomen but differs in possessing an areolated epithelium extending from the peristomium to chaetiger 3, a distinct constriction between the thorax and abdomen, and a short prostomial palpode. Multiple gene markers and methyl green staining patterns are provided for both species. Notably, *B. multibranchia***sp. nov**. exhibited intraspecific divergence in COI sequences among collection localities.

## Introduction

Members of the family Capitellidae Grube, 1862 are common inhabitants of marine infaunal communities, where they function as subsurface deposit feeders and play a significant role in nutrient cycling (Jumars et al. 2015). This family is widely distributed across a broad depth range, from intertidal to abyssal zones (Magalhães and Blake 2020) and tolerates a wide salinity range from brackish to euhaline waters. The family currently comprises over 200 described species across 42 genera worldwide (Read and Fauchald 2026), with the majority (21 genera) being monotypic (Hernández-Alcántara et al. 2022). Among these, *Barantolla* Southern, 1921 and *Parheteromastus* Monro, 1937 are two small genera: *Barantolla* includes five accepted species, whereas *Parheteromastus* remains monospecific. The latter genus is especially poorly documented globally. Its sole species, *Parheteromastus tenuis* Monro, 1937, was originally described from Maungmagan, Myanmar, and was subsequently reported from Mozambique by Day (1967). More recently, Lin et al. (2014) reported *P. tenuis* from mangrove wetlands of Fujian Province, China. Presently, accurate species identification within Capitellidae remains challenging because the external morphology is relatively simple and several diagnostic characters, such as the distribution of capillary chaetae on the thorax, are known to vary ontogenetically (Blake 2000; Magalhães and Blake 2020). Consequently, integrative analyses incorporating both morphological and molecular data are essential for resolving species boundaries in this family.

In China, the family Capitellidae remains poorly studied, with only a few taxa reported to date. Yang and Sun (1988) provided the first taxonomic review of this family, documenting five species belonging to four genera; however, most of these species were originally described from European or North American waters, and their identities require verification. According to an updated species list compiled by Li and Gan (2022), 17 species in 10 genera have been recorded from Chinese waters, yet the genus *Barantolla* was not included. In the present study, extensive sampling along the southern coasts of China during recent ecological surveys yielded two new species belonging to the genera *Barantolla* and *Parheteromastus*. These species are formally described and illustrated herein based on integrated morphological and molecular analyses. This study represents a further contribution to capitellid taxonomy, aiming to bridge the gap in taxonomic knowledge of this family in Chinese waters.

## Material and methods

### Field sampling and morphological observations

**Figure 1.**
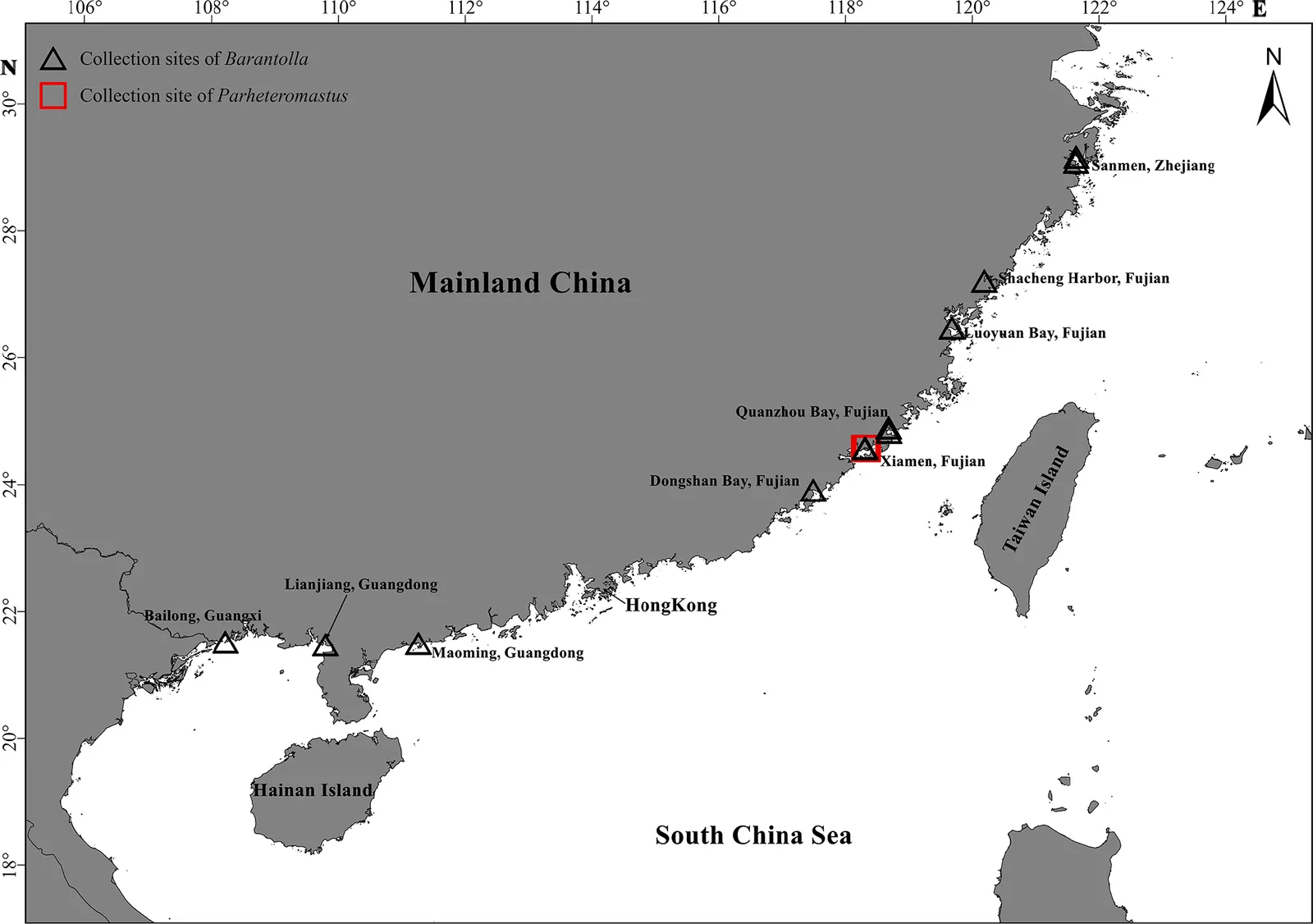
Map of collection sites of *Barantolla multibranchia* sp. nov. (triangles) and *Parheteromastus simplex* sp. nov. (square).

Sampling was conducted along the southern coasts of China (Fig. 1), spanning from Zhejiang Province in the east to Guangxi Province in the west, during 2019–2026. Sediment samples were collected using a 0.05 m^2^ grab sampler during high tide and subsequently washed through a 0.5 mm sieve in the field. Specimens retained on the sieve were manually picked and fixed in 100% ethanol. Morphological observations and photographs were taken using a Leica M205A stereomicroscope equipped with a DFC 550 digital camera and a Leica DM6B compound microscope. For SEM observation, samples were dehydrated using a graded ethanol series, followed by critical-point drying and gold-palladium sputter coating, and then observed under a TESCAN MIRA microscope. The methyl green staining pattern (MGSP) was used to identify the distribution of glandular areas. All specimens examined in this study were deposited at the Third Institute of Oceanography, Ministry of Natural Resources, Xiamen, China (TIO, MNR).

### DNA extraction, PCR amplification and sequencing

Tissue samples for DNA extraction were obtained from the specimens listed in Table 1 using a Transgen Micro Genomic DNA EE 181 Kit (Transgen, Beijing, China) following the manufacturer’s protocol. Polymerase chain reactions (PCRs) were performed to amplify five partial gene fragments: two mitochondrial genes (COI and 16S rRNA) and three nuclear genes (18S rRNA, 28S rRNA, and H3). The following primer pairs were used: LCO1490 and HCO2198 (Folmer et al. 1994) and polyLCO and polyHCO (Carr et al. 2011) for COI; Ann16SF and 16SbrH (Palumbi 1996; Sjölin et al. 2005) for 16S rRNA; 18SA and 18SB (Medlin et al. 1988; Nygren and Sundberg 2003) for 18S rRNA; NLF184/21 and D3aR (Lenaers et al. 1989; Van der Auwera et al. 1994) for 28S rRNA; and H3aF and H3aR (Colgan et al. 1998) for H3. The successful PCR products were bidirectionally sequenced on an ABI 3730XL DNA analyzer at Sangon Biotech (Shanghai, China). Both forward and reverse reads were manually assembled into consensus sequences using DNAMAN software (Lynnon Biosoft, Quebec, Canada).

**Table 1.** Information for the examined specimens of *Barantolla multibranchia* sp. nov. and *Parheteromastus simplex* sp. nov. Abbreviation: *N*: number of individuals.

**Species**	**Voucher**	**Location**	**Substrate**	**Date**	**Lat., long**.	**COI**	**16S**	**18S**	**28S**	**H3**	***N***
*B. multibranchia* sp. nov.	TIO-Poly 155	Sanmen, Zhejiang	Intertidal mud	26 Dec. 2022	29.09°N, 121.63°E	—	PZ486615	PZ486637	PZ486626	PZ499943	1
	TIO-Poly 156	Sanmen, Zhejiang	Intertidal mud	31 Mar. 2020	29.17°N, 121.64°E	PZ487945	PZ486616	PZ486638	PZ486627	PZ499944	12
	TIO-Poly 157	Shacheng Harbor, Fujian	Intertidal mud	30 May 2025	27.21°N, 120.19°E	—	PZ486625	PZ486639	PZ486635	PZ499952	2
	TIO-Poly 158	Luoyuan Bay, Fujian	Intertidal mud	10 Jan. 2026	26.48°N, 119.68°E	PZ487947	PZ486619	PZ486642	PZ486630	PZ499947	2
	TIO-Poly 153*	Quanzhou Bay, Fujian	Muddy sand; 2 m	26 Sep. 2022	24.83°N, 118.68°E	—	—	—	—	—	1
	TIO-Poly 154**	Quanzhou Bay, Fujian	Muddy sand; 2 m	26 Sep. 2022	24.83°N, 118.68°E	PZ487946	PZ486617	PZ486640	PZ486628	PZ499945	4
	TIO-Poly 159**	Quanzhou Bay, Fujian	Mud; 2 m	18 May 2022	24.90°N, 118.68°E	—	PZ486618	PZ486641	PZ486629	PZ499946	5
	TIO-Poly 160**	Xiamen, Fujian	Intertidal mud	16 Mar. 2025	24.57°N, 118.30°E	PZ487951	PZ486624	PZ486646	PZ486634	PZ499951	4
	TIO-Poly 161**	Xiamen, Fujian	Intertidal mud	29 Jan. 2024	24.58°N, 118.31°E	PZ487952	PZ486623	PZ486647	PZ486636	PZ499953	4
	TIO-Poly 162	Dongshan Bay, Fujian	Intertidal mud	24 Feb. 2019	23.92°N, 117.49°E	**—**	**—**	PZ486648	—	PZ499954	1
	TIO-Poly 163	Maoming, Guangdong	Mangrove; mud	2 Nov. 2025	21.50°N, 111.27°E	PZ487948	PZ486620	PZ486643	PZ486631	PZ499948	1
	TIO-Poly 164	Lianjiang, Guangdong	Mangrove; Muddy sand	13 May 2023	21.47°N, 109.80°E	PZ487949	PZ486621	PZ486644	PZ486632	PZ499949	4
	TIO-Poly 165	Bailong, Guangxi	Intertidal mud	15 May 2023	21.52°N, 108.22°E	PZ487950	PZ486622	PZ486645	PZ486633	PZ499950	1
*P. simplex* sp. nov.	TIO-Poly 166*	Xiamen, Fujian	Intertidal mud	16 Mar. 2025	24.57°N, 118.30°E	—	—	—	—	—	1
	TIO-Poly 167**	Xiamen, Fujian	Intertidal mud	16 Mar. 2025	24.57°N, 118.30°E	PZ486392	PZ486651	PZ486649	PZ486653	PZ499941	1
	TIO-Poly 168**	Xiamen, Fujian	Intertidal mud	11 Mar. 2020	24.57°N, 118.34°E	PZ486393	PZ486652	PZ486650	PZ486654	PZ499942	1

Note: *, holotype; **, paratype.

### Sequences analysis

The protein-coding sequences (COI and H3) were aligned using the MUSCLE algorithm (Edgar 2004), while ribosomal RNA sequences (16S, 18S, and 28S rRNA) were aligned using MAFFT 7 (Katoh and Standley 2013), both with default settings. The intraspecific and interspecific genetic distances were calculated based on the Kimura 2-parameter (K2P) model (Kimura 1980) implemented in MEGA X (Kumar et al. 2018).

## Taxonomy

### Class Polychaeta Grube, 1850

**Family Capitellidae Grube, 1862**

#### Genus Barantolla

Taxon classificationAnimaliaPolychaetaCapitellidae

Southern, 1921

03CF3E4E-7D17-52D9-A409-AB83602CB4AE

##### Type species.

*Barantolla sculpta* Southern, 1921

##### Generic diagnosis.

(amended after Magalhães and Blake 2020) Prostomium conical, palpode present or absent; eyespots absent, nuchal organs present. Peristomium clearly distinct from prostomium; achaetous segment absent. First chaetiger uniramous or biramous. Eleven thoracic chaetigers. Chaetigers 1–6 with capillaries; one transitional thoracic chaetiger may be present on chaetiger 6 or chaetiger 7; remaining thoracic chaetigers with notopodial and neuropodial hooks. Thorax clearly demarcated from abdomen. Abdominal segments with hooded hooks in both rami, different in shape and size than thoracic hooks. Branchiae present or absent; when present, branched and dorsal. Genital pores not observed. Lateral organs present on thorax. Pygidium may be adorned with ventral cirrus.

##### Remarks.

The current generic diagnosis of *Barantolla* encompasses interspecific variation in chaetal details. Most species bear only capillaries on the first six chaetigers and hooks on subsequent thoracic chaetigers, while *B. americana* uniquely possesses mixed chaetae on chaetiger 7. The new species differs in having mixed chaetae in both rami on chaetiger 6 and a short palpode at the prostomial tip, which is not seen in congeneric species. To accommodate these new features, we have expanded the generic diagnosis.

#### Barantolla multibranchia sp. nov.

Taxon classificationAnimaliaPolychaetaCapitellidae

7192603A-349E-59CA-904C-EC3FA7C2605A

https://zoobank.org/C24543E7-83C7-45A4-9BA8-4456BD649BDA

Figs 2A–R, 3A–I

##### Material examined.

***Holotype***: China • TIO-Poly 153, complete; Quanzhou Bay; sta. Q18; 24.83°N, 118.68°E; 2 m depth, muddy sand; 23 Sep. 2022; Jun-Hui Lin leg. ***Paratypes***: China • TIO-Poly 154, 4 specs., complete or incomplete; same site as holotype; one used for DNA extraction. • TIO-Poly 159, 5 specs, incomplete; Quanzhou Bay; sta. Q7; 24.90°N, 118.68°E; 2 m depth, mud; 18 May 2022; Jun-Hui Lin leg. • TIO-Poly 160, 4 specs, complete or incomplete; Xiamen Bay; 24.57°N, 118.30°E; intertidal mud; 16 May 2025; Jun-Hui Lin leg. • TIO-Poly 161, 4 specs, incomplete; Xiamen Bay; 24.58°N, 118.31°E; intertidal mud; 29 Jan. 2024; Jun-Hui Lin leg.

**Figure 2.**
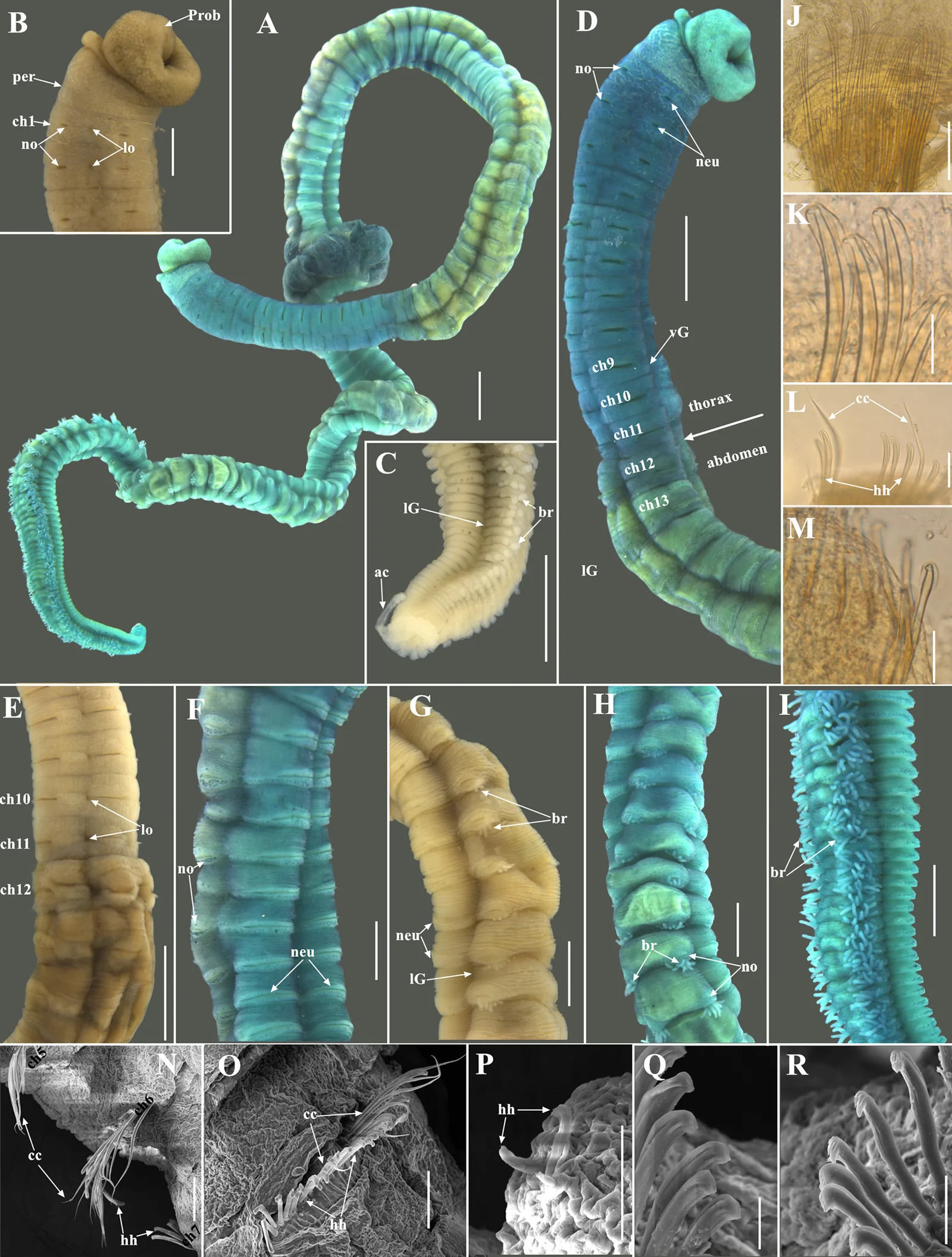
Light and SEM micrographs **(N–R**) of *Barantolla multibranchia* sp. nov., holotype (**A–I**) and paratype (**J–R**). **A**. Entire body showing MGSP; **B**. Anterior end in lateral view; **C**. Posterior end in lateral view; **D**. Anterior 17 chaetigers in lateral view; **E**. Transition between thorax and abdomen in dorso-lateral view; **F**. Middle abdomen in ventro-lateral view; **G**. Middle-posterior abdomen in dorso-lateral view; **H**. Posterior abdomen in dorsal view; **I**. Posterior abdomen near pygidium in dorso-lateral view; **J, K**. Long-handled hooks at chaetiger 7; **L**. Mixed chaetae in neuropodium of chaetiger 6; **M**. Hooded hooks in middle abdomen; **N**. Notopodia of chaetigers 5–7; **O**. Neuropodium of chaetiger 6; **P**. Hooded hooks near pygidium; **Q**. Long-handled hooded hooks in thorax; **R**. Abdominal hooded hooks in middle abdomen. Abbreviations: ac = anal cirrus, br = branchia, cc = capillary chaetae, ch = chaetiger, hh = hooded hook, lG = lateral groove, lo = lateral organ, neu = neuropodium, no = notopodium, per = peristomium, prob = proboscis, vG = ventral groove. Scale bars: 1 mm (**A, D, E**); 0.5 mm (**B, C, F–I**); 50 μm (**J, L, N, O**); 20 μm (**K, M, P**); 10 μm (**Q, R**).

**Figure 3.**
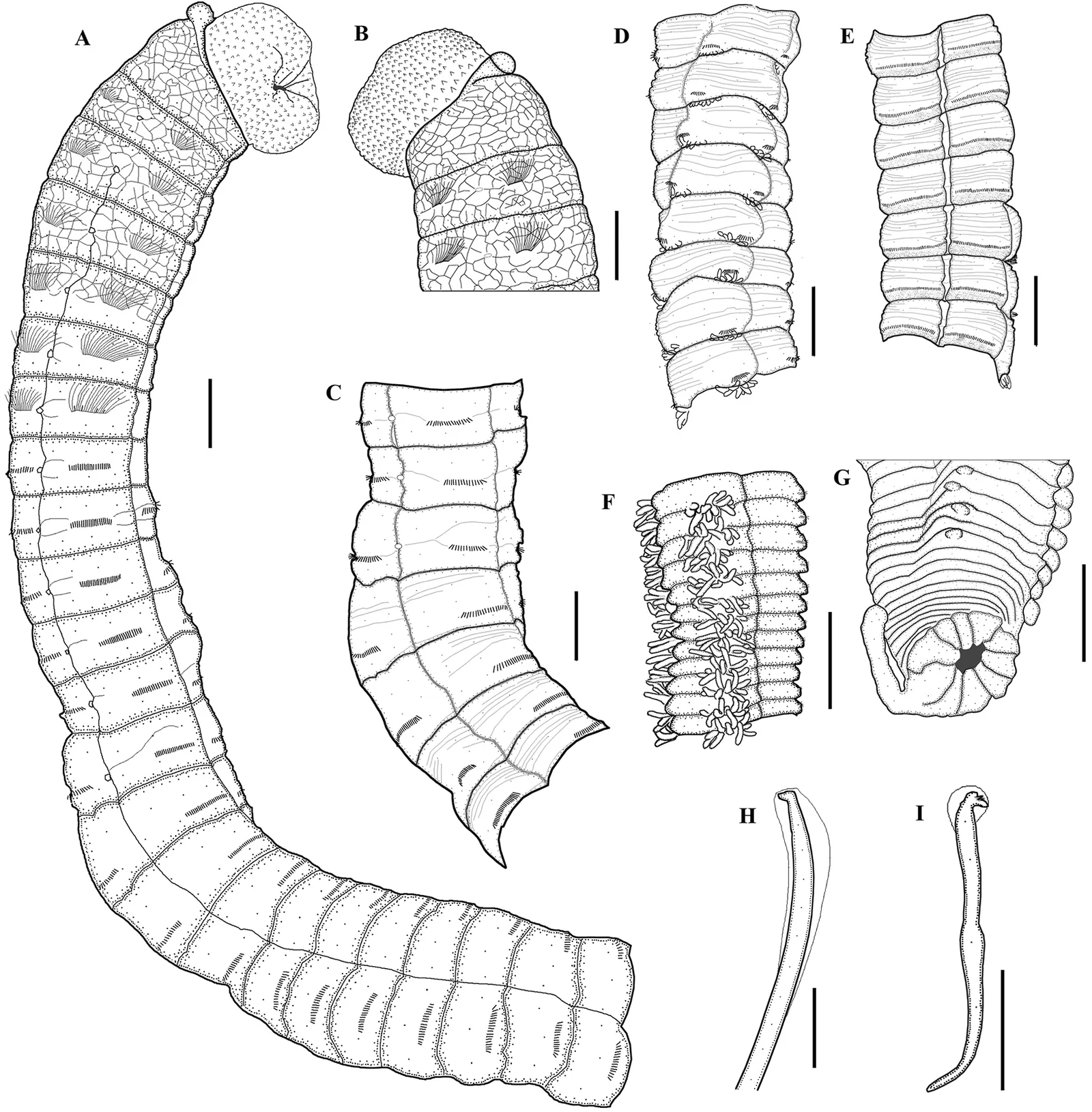
*Barantolla multibranchia* sp. nov., holotype. **A**. Anterior 22 chaetigers in lateral view; **B**. Anterior end in lateral view; **C**. Chaetigers 10–16 in ventro-lateral view; **D**. Middle-posterior abdomen in dorsal view; **E**. Posterior abdomen in ventral view; **F**. Crowded abdominal segments in dorso-lateral view; **G**. Posterior end with anal cirrus in dorso-lateral view; **H**. Thoracic hook; **I**. Abdominal hook. Scale bars: 0.5 mm (**A–F**); 200 μm (**G**); 20 μm (**H, I**).

##### Non-type material.

China • TIO-Poly 155, 1 spec., incomplete; Sanmen Bay; 29.09°N, 121.63°E; intertidal mud; 26 Dec. 2022; Wen-Sheng Lin leg. • TIO-Poly 156, 12 specs, complete or incomplete; Sanmen Bay; 29.17°N, 121.64°E; intertidal mud; 31 Mar. 2020; Wen-Sheng Lin leg. • TIO-Poly 157, 2 specs, incomplete; Shacheng Harbor; 27.21°N, 120.19°E; intertidal mud; 30 May 2025; Wen-Sheng Lin leg. • TIO-Poly 158, 2 specs, incomplete; Luoyuan Bay; 26.48°N, 119.68°E; intertidal mud; 10 Jan. 2026; Wen-Sheng Lin leg. • TIO-Poly 162, 1 spec., incomplete; Dongshan Bay; 23.92°N, 117.49°E; intertidal mud; 24 Feb. 2019; Wen-Sheng Lin leg. • TIO-Poly 163, 1 spec., incomplete; Maoming; 21.50°N, 111.27°E; mangrove, mud; 2 Nov. 2025; Wen-Sheng Lin leg. • TIO-Poly 164, 4 specs, incomplete; Lianjiang; 21.47°N, 109.80°E; mangrove, muddy sand; 13 May 2023; Wen-Sheng Lin leg. • TIO-Poly 165, 1 spec., incomplete; Bailong; 21.52°N, 108.22°E; intertidal mud; 15 May 2023; Wen-Sheng Lin leg.

##### Description.

Holotype complete (Fig. 2A), measuring 47.78 mm long by 1.20 mm wide (widest at chaetiger 13) for about 173 chaetigers. Paratypes complete or incomplete, ranging from 18.64–36.13 mm long, 0.92–1.15 mm wide for 47–152 chaetigers. Body nearly cylindrical, widest in anterior abdomen. Colour in alcohol tan (Fig. 2B). Epithelium clearly areolated from peristomium to chaetiger 3, and smooth on following segments (Figs 2D, 3A). Abdominal segments with transverse wrinkles (Fig. 2G). Ventral and lateral grooves present from anterior thorax (Fig. 2A, D). Nuchal organs not observed.

Prostomium conical with short palpode (Figs 2B, 2D, 3B). Proboscis everted in holotype, covered with numerous minute papillae (Fig. 2B, D). Eyespots absent. Peristomium achaetous, separated from chaetiger 1, 2 times wider than long, longer than chaetiger 1.

Thorax consisting of achaetous peristomium and 11 chaetigers (Figs 2A, 2D, 3A); intersegmental grooves distinct throughout. Lateral organs located between noto- and neuropodia, closer to notopodia, as small pores (Fig. 2D, E). Genital pores not observed. First chaetiger biramous, 2.0 times wider than long (Fig. 2B, D). Following segments 1.5–2.5 times wider than long. Chaetigers 1–5 with only capillaries in both rami, 14–24 per fascicle in notopodia and 16–30 per fascicle in neuropodia. Chaetiger 6 with mixed chaetae in both rami (Fig. 2L, N, O), around 21 per fascicle in notopodia and 26 per fascicle in neuropodia. Chaetigers 7–11 with only long-handled hooks (Fig. 2J, K, Q), 18–30 per fascicle in notopodia and 20–34 per fascicle in neuropodia. Notopodia inserted dorso-laterally, moving to dorsal side gradually, and neuropodia ventro-laterally. Chaetal fascicles inserted near midline of thoracic segments.

Transition between thorax and abdomen marked by abrupt broadening of anterior abdominal segments and chaetal change (Figs 2D, 2E, 3C). Abdominal segments longer and wider than last thoracic chaetigers in anterior abdomen (Fig. 2E), tapering gradually towards pygidium (Fig. 2C). Notopodial lobes well separated in anterior abdomen. Elevated tori pad forming a thin membrane surrounding the segment like a collar in posterior abdomen (Fig. 2G). Posterior abdominal segments crowded (Figs 2I, 3F). Abdominal chaetigers with hooded hooks throughout, with 24–40 hooks per fascicle in anterior abdomen, increasing to 50–80 hooks in posterior abdomen, decreasing to 2 or 3 hooks in posterior end (Fig. 2P). In anterior abdomen, notopodial lobes dorsally located and neuropodial lobes ventro-lateral. From middle abdomen, notopodial lobes dorsal and neuropodial lobes ventral (Figs 2F–H, 3D, 3E). Chaetal fascicles positioned posterior to midsegment in anterior abdomen, and near posterior edge of segment toward the pygidium (Fig. 2H).

Thoracic long-handled hooks without angled node, distal 1/6 to 1/5 hooded (Fig. 2J); hoods around 5 times longer than wide (Figs 2K, 3H). Abdominal hooded hooks much shorter than thoracic ones, with angled node, developed shoulder, posterior shaft slightly longer than anterior one, attenuated at terminal end (Figs 2M, 3I). Hood slightly longer than wide. Hooded hooks with 3 rows of teeth above main fang (Fig. 2R): 3 teeth in basal row, 3 teeth in middle row, 2 teeth in apical row (smaller than in basal row). Main fang subtriangular, longer than wide.

Dorsal branchiae commencing from around chaetiger 74 in middle abdomen, located behind notopodial tori. Anterior branchiae consisting of 2 or 3 nipple-like lobes (Fig. 2G). Towards the tail branchial lobes increasing in number and length, the largest with 14 finger-like lobes (Figs 2H, 2I, 3F). Near pygidium branchial lobes decreasing to 1 or 2 spherical lobes (Figs 2C, 3G).

Pygidium adorned with a mid-ventral cirrus (Figs 2C, 3G).

##### Methyl green staining pattern.

(Figs 2A, 2D, 2F, 2H) Chaetigers 1–4 stained with medium green. Abdominal segments with sparse green speckles on areas behind parapodial tori.

##### DNA sequences.

Twelve specimens of *B. multibranchia* sp. nov. from nine sampling localities were sequenced in this study. This yielded eight partial COI sequences (PZ487945–PZ487952), 11 partial 16S sequences (PZ486615–PZ486625), 12 partial 18S sequences (PZ486637–PZ486648), 11 partial 28S sequences (PZ486626–PZ486636), and 12 partial H3 sequences (PZ499943–PZ499954).

##### Distribution.

Widely distributed along the southern coasts of China, spanning from Zhejiang Province in the east to Guangxi Province in the west.

##### Ecology.

The new species mainly inhabits intertidal or shallow waters in estuarine areas or inner bays, where the sediment consists of mud, muddy sand, or sandy mud.

##### Etymology.

The specific epithet multibranchia is composed of the Latin prefix *multi-* (many) and the Latin noun *branchia* (gills), referring to the presence of numerous branchiae in the new species. It is used as a noun in apposition and is indeclinable.

##### Remarks.

*Barantolla multibranchia* sp. nov. is assigned to the genus *Barantolla* based on the presence of an achaetous peristomium and 11 thoracic chaetigers, with the first six chaetigers bearing capillary chaetae. Since Southern (1921) erected the genus, five species have been formally described worldwide (Hartman 1963; Hutchings 1974; Yabe and Mawatari 1998; Çinar et al. 2022). Among these, only *B. sculpta*, described from a brackish lake near Calcutta, India, has been reported to bear dorsal branchiae on the abdomen. The new species closely resembles *B. sculpta* in sharing dorsal branchiae on the abdomen, an areolated epithelium on the anterior thorax, and a complete chaetiger 1. However, it differs from the latter in details of the chaetae at chaetiger 6 and in epithelial texture. *Barantolla multibranchia* sp. nov. has mixed chaetae in both rami of chaetiger 6 and lacks the thoracic sculpturing. Whereas the unique sculpturing of the thorax was characteristic of *B. sculpta*, which possesses only capillaries on the first six thoracic chaetigers. Owing to the absence of a detailed description of hooded hooks in the original reference, a comparison of this character is not possible.

##### Genetic divergence.

Within *B. multibranchia* sp. nov. the intraspecific K2P genetic distances among specimens sequenced in this study varied across gene markers (Suppl. material 1): COI (0–4.3%), 16S(0–0.9%), 18S(0%), 28S(0–0.1%), and H3(0–1.6%). Notably, the COI-based genetic distances between specimens from Guangxi and those from other localities are substantially higher, suggesting that the Guangxi population may be undergoing divergence.

The Hainan specimen (accession number PQ010756) exhibits a K2P genetic distance of 1.3% from the paratype of *B. multibranchia* sp. nov. (PZ487946), a value well within the intraspecific range, indicating that the Hainan specimen belongs to the new species described herein. The COI-based genetic distance between *B. multibranchia* sp. nov. and other species with available molecular sequences ranged from 22.3% to 44.6%, greater than the intraspecific genetic distance (Table 2). Additionally, the 18S-based K2P genetic distance between *B. lepte* Hutchings, 1974 (AB106265) and *B. multibranchia* sp. nov. is 1.2%, which exceeds the intraspecific divergence (0%) in *B. multibranchia*, confirming the distinction of the new species from *B. lepte*.

**Table 2.** The COI-based genetic divergence (K2P) between *Barantolla* species with the available sequences.

**No**.	**Species (529 bp)**	**Location**	**1**	**2**	**3**	**4**
1	*Barantolla* sp. 1 ST2018 (LC208116)	Hokkaido, Japan				
2	*Barantolla* sp. 2 ST2018 (LC208117)	Okinawa, Japan	0.451			
3	*Barantolla americana* (HM473728)	Bering Sea	0.243	0.434		
4	*Barantolla* sp. HN (PQ010756)	Hainan, China	0.228	0.446	0.237	
5	*Barantolla multibranchia* sp. nov. (PZ487946)	Fujian, China	0.223	0.439	0.237	0.013

#### Genus Parheteromastus

Taxon classificationAnimaliaPolychaetaCapitellidae

Monro, 1937

A73C99EA-4F21-592D-AD69-A07E172EA4E2

##### Type species.

*Parheteromastus tenuis* Monro, 1937.

##### Diagnosis.

(amended after Magalhães and Blake 2020) Prostomium conical, eyespots absent. Peristomium clearly distinct from prostomium; achaetous segment absent. First chaetiger biramous. Eleven thoracic chaetigers. Chaetigers 1–4 with only capillaries and chaetigers 5–11 with only hooded hooks. Thorax may or may not be clearly demarcated from abdomen. Abdominal segments with only hooded hooks. Branchiae absent. Genital pores and lateral organs not described. Pygidium adorned with a single short cirrus.

#### Parheteromastus simplex sp. nov.

Taxon classificationAnimaliaPolychaetaCapitellidae

611437D0-905B-5DDC-A5F2-9E958148FB9E

https://zoobank.org/7A0B9202-2446-477E-BDED-3DB513623D39

Figs 4A–J, 5A–F

##### Material examined.

***Holotype***. China • TIO-Poly165, complete; intertidal zone near Dadeng Island; 24.57°N, 118.30°E; intertidal mud; 16 March 2025; Jun-Hui Lin leg. ***Paratypes***. China • TIO-Poly166, 1 spec., incomplete; same site as TIO-Poly165. China • TIO-Poly167, 1 spec., incomplete; intertidal zone near Dadeng Island; 24.57°N, 118.34°E; intertidal mud; 11 March 2020; Jun-Hui Lin leg.

**Figure 4.**
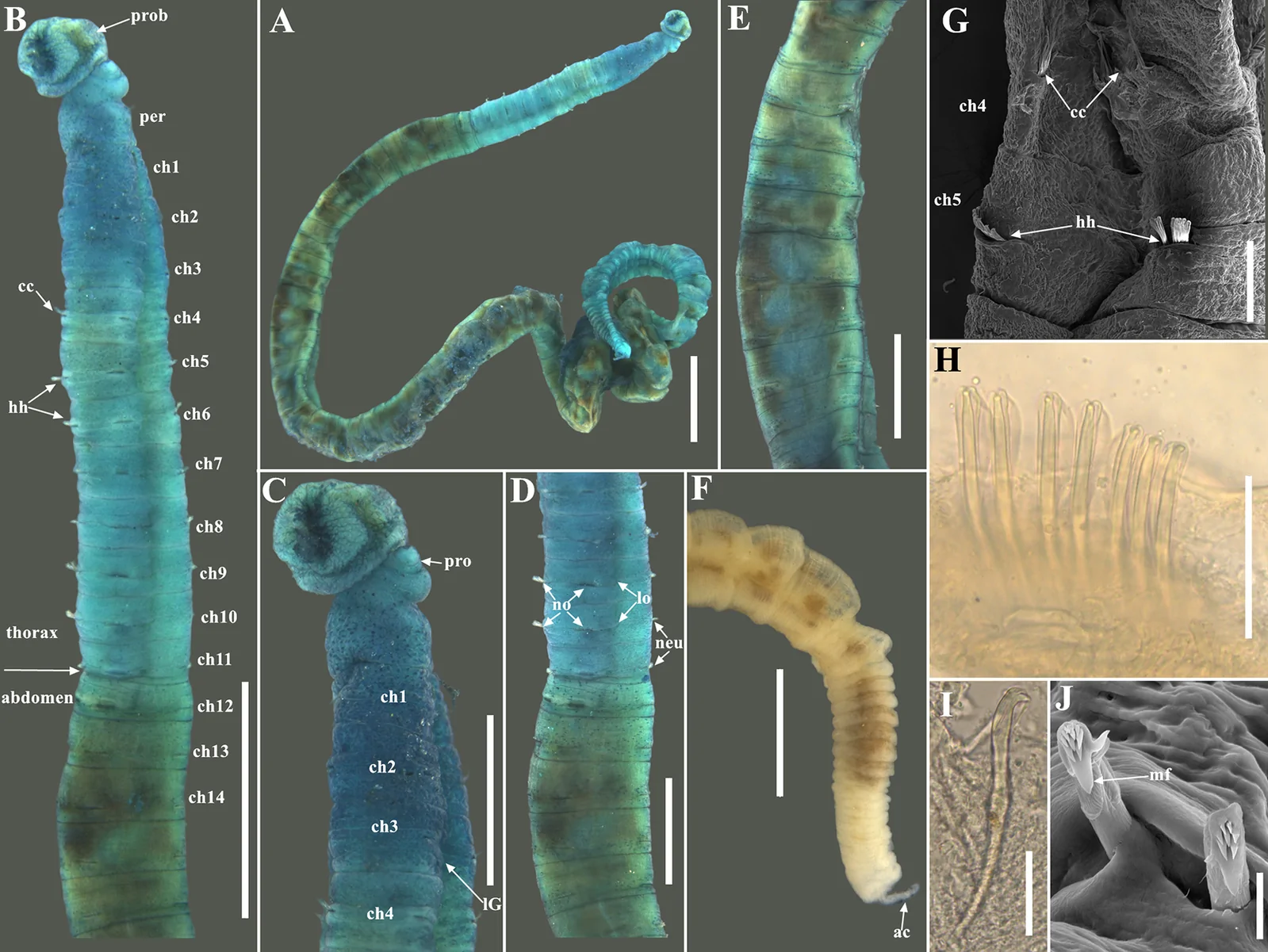
Light and SEM micrographs (**G, J**) of *Parheteromastus simplex* sp. nov., holotype (**A–F**) and paratype (**G–J**). **A**. Entire body showing MGSP; **B**. Anterior 16 chaetigers in dorso-lateral view; **C**. Anterior end in dorso-lateral view; **D**. Transition between thorax and abdomen in lateral view; **E**. Anterior abdomen in lateral view; **F**. Posterior abdomen with anal cirrus in lateral view; **G**. Chaetigers 4–5 in dorsal view; **H**. Hooded hooks at chaetiger 6; **I, J**. Hooded hooks in middle abdomen. Abbreviations: ac = anal cirrus, cc = capillary chaetae, ch = chaetiger, hh = hooded hook, lo = lateral organ, mf = main fang, neu = neuropodia, no = notopodia, per = peristomium, pro = prostomium, prob = proboscis. Scale bars: 1 mm (**A–B**); 0.5 mm (**C–F**); 100 μm (**G–H**); 20 μm (**I**); 5 μm (**J**).

**Figure 5.**
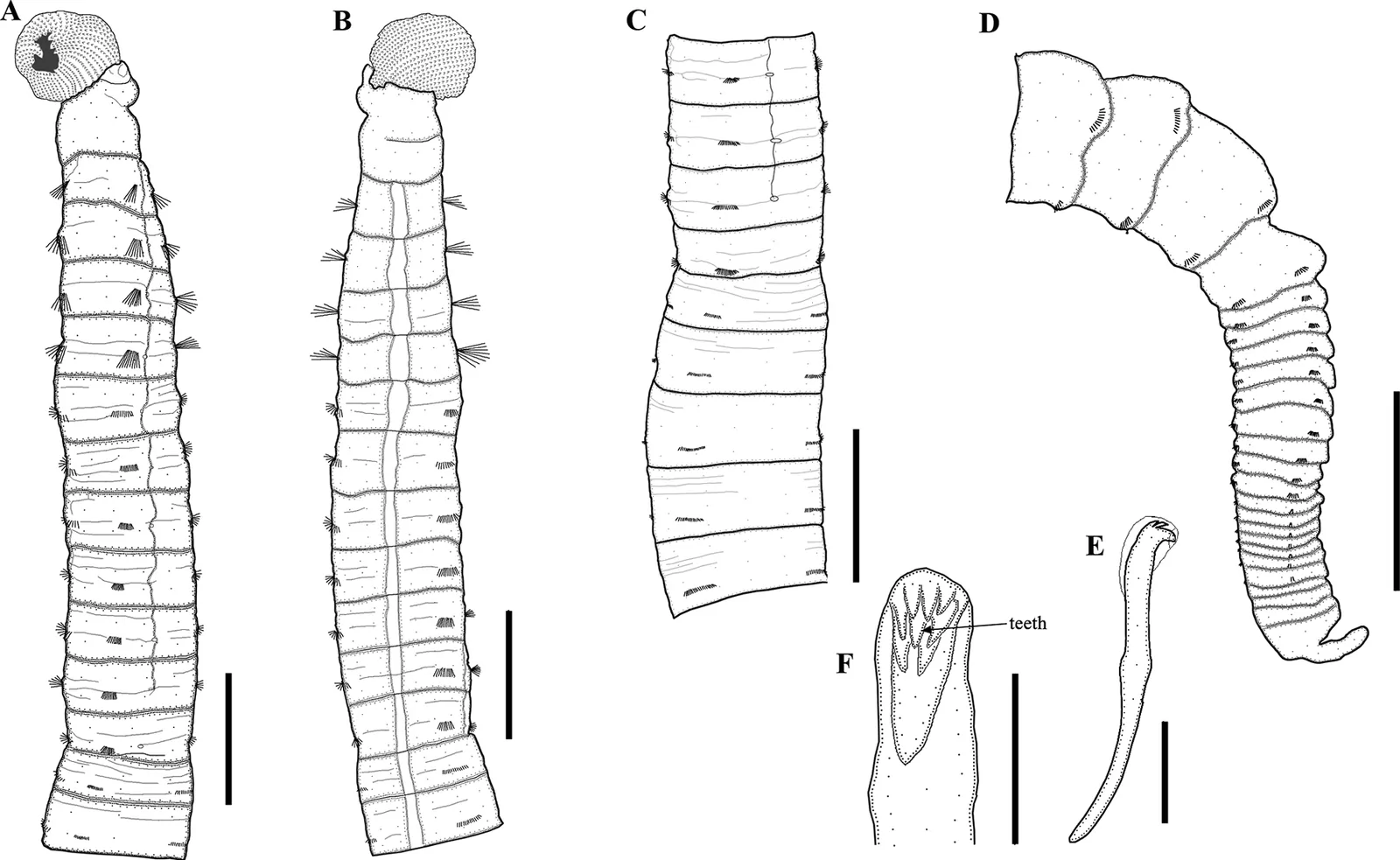
*Parheteromastus simplex* sp. nov., holotype (**A–D**) and paratype (**E, F**). **A**. Anterior 13 chaetigers in dorso-lateral view; **B**. Anterior 13 chaetigers in ventral view; **C**. Chaetigers 8–16 in lateral view; **D**. Posterior abdomen in lateral view; **E, F**. Abdominal hooded hook. Scale bars: 0.5 mm (**A–D**); 20 μm (**E**); 5 μm (**F**).

##### Description.

Holotype complete with posterior part heavily coiled (Fig. 4A), measuring 20.11 mm long by 0.55 mm wide (at chaetiger 14) for about 104 chaetigers. Paratypes incomplete, ranging from 13.48–22.83 mm long, 0.56–0.61 mm wide for 47–52 chaetigers. Body thread-like, widest in anterior abdomen. Lateral and ventral grooves present in thorax. Colour in alcohol tan. Nuchal organ not observed.

Prostomium conical with short palpode, eyespots absent. Proboscis everted in holotype, covered with numerous minute papillae (Figs 4A–C, 5A, 5B). Peristomium achaetous, longer than wide, longer than chaetiger 1. Peristomium clearly demarked from chaetiger 1 (Fig. 4B, C).

Thorax with achaetous peristomium and 11 chaetigers in holotype; thoracic chaetigers biannulate; first chaetiger biramous (Fig. 4B). Thoracic segments wider than long, with epithelium areolated from peristomium up to chaetiger 3 (Fig. 4C), and smooth on following segments (Fig. 4D). Intersegmental grooves distinct. Lateral organs located between noto- and neuropodia, closer to notopodia, as small pores (Fig. 4D). Genital pores not observed. First chaetiger 1.2 times wider than long; following segments 1.3–2.2 times wider than long. Chaetigers 1–4 with only capillaries in both rami, 5–10 per fascicle in notopodia and 6–10 per fascicle in neuropodia. Chaetigers 5–11 with only long-handled hooks (Fig. 4G), 8–10 per fascicle in notopodia and 8–12 per fascicle in neuropodia. Notopodia inserted dorso-laterally, moving to dorsal side posteriorly; neuropodia inserted ventro-laterally throughout. Chaetal fascicles inserted near midline of thoracic segments.

Transition between thorax and abdomen marked by constriction and chaetal change (Figs 4D, 5C). Abdominal segments longer and wider than last thoracic chaetiger in anterior abdomen, tapering gradually towards pygidium (Fig. 4F). Parapodial lobes reduced, well separated in entire abdomen (Fig. 4D, E). Abdominal chaetigers with hooded hooks throughout, with about 10 hooks per fascicle in abdomen, decreasing to 1 or 2 hooks per fascicle in posterior end. Notopodial lobes located dorsally and neuropodial lobes ventral. Chaetal fascicles positioned posterior to mid-segment in anterior abdomen (Fig. 4D), and near posterior edge of segment toward the pygidium (Fig. 4E).

Thoracic hooded hooks without angled node, distal 1/6 to 1/5 hooded; hoods about 5 times longer than wide (Fig. 4H). Abdominal hooded hooks much shorter than thoracic ones, with angled node, developed shoulder, posterior shaft slightly longer than anterior one, attenuated at terminal end (Figs 4I, 5E). Hood slightly longer than wide. Hooded hooks with 3 rows of teeth above main fang (Figs 4J, 5F): 2 teeth in basal row, 3 teeth in middle row, 3 teeth in apical row. Main fang subtriangular, longer than wide.

Branchiae absent. Pygidium adorned with a mid-ventral cirrus (Figs 4F, 5D).

##### Methyl green staining pattern.

(Fig. 4A–E) Anterior three chaetigers stained with dense green speckles. Sparse dark spots of stain scattered on anterior two abdominal chaetigers, and on posterior quarter of each following segment.

##### DNA sequences.

Two *Parheteromastus* specimens collected from two sampling sites were sequenced, and all five gene fragments were successfully obtained. No intraspecific genetic variation was detected between the two specimens across all markers, except for a single nucleotide substitution in the COI gene. These sequences represent the first molecular data for species of the genus *Parheteromastus*.

##### Distribution.

Currently known from the intertidal zones near Dadeng Island, Xiamen Bay, and possibly present in the mangrove wetlands of Fujian Province.

##### Ecology.

The new species inhabits intertidal sediment characterized by mud, muddy sand, or sandy mud.

##### Etymology.

This species was named after its plain external morphology: the Latin adjective referring to a plain state.

##### Remarks.

*Parheteromastus* was initially erected by Monro (1937) based on specimens collected from the coasts of Myanmar. To date, species of *Parheteromastus* have been sparsely documented worldwide. Prior to this study, the genus was considered monotypic, with only *Parheteromastus tenuis* having been described. The new species described herein agrees well with the generic diagnosis: its thorax has an achaetous peristomium and 11 chaetigers, of which the anterior four chaetigers possess capillaries and the remaining seven chaetigers bear only long-handled hooks. *Parheteromastus simplex* sp. nov. is similar to *P. tenuis* in several respects: both species are small worms with reduced abdominal parapodial tori, lack branchiae, and possess a short cirrus on the pygidium. However, the two species differ in the appearance of the anterior thorax and the transition of the thorax and abdomen. *Parheteromastus simplex* sp. nov. has an areolated epithelium from the peristomium to chaetiger 3, and the transition between the thorax and abdomen is clearly demarcated by a constriction. In contrast, *P. tenuis* has a smooth thorax and the transition is indistinct. With respect to the dental formula, no description or illustration of abdominal hooks is available for *P. tenuis*, which prevents a detailed comparison between the two species.

## Supplementary Material

XML Treatment for Barantolla

XML Treatment for Barantolla multibranchia

XML Treatment for Parheteromastus

XML Treatment for Parheteromastus simplex

## Additional information

### Artificial Intelligence (AI) use

The authors accept full responsibility for the content of the manuscript, including the disclosure of any use of AI.

No AI tools were used in the preparation of this manuscript.

### Data availability

All of the data that support the findings of this study are available in the main text or Supplementary Information.

## Supplementary materials

Supplementary material 1 Molecular data Data type xlsx Explanation note **table SS1**. Intraspecific divergence of COI, 16S, 28S and H3 of *Barantolla multibranchia* specimens in this study. https://binary.pensoft.net/file/1776227This dataset is made available under the Open Database License (http://opendatacommons.org/licenses/odbl/1.0/). The Open Database License (ODbL) is a license agreement intended to allow users to freely share, modify, and use this Dataset while maintaining this same freedom for others, provided that the original source and author(s) are credited. Jun-Hui Lin, María E. García-Garza, Shu-Yi Zhang, He-Shan Lin
